# *Sesuvium portulacastrum* SpC3H Enhances Salt Tolerance of *Arabidopsis thaliana* by Regulating Lignin Synthesis and Scavenging Reactive Oxygen Species

**DOI:** 10.3390/plants14213347

**Published:** 2025-10-31

**Authors:** Yuxin Li, Yanping Hu, Tingting Zhang, Yushan Wang, Zhiguang Sun, Yang Zhou

**Affiliations:** 1 Key Laboratory for Quality Regulation of Tropical Horticultural Crops of Hainan Province, School of Tropical Agriculture and Forestry (School of Agricultural and Rural Affairs, School of Rural Revitalization), Hainan University, Haikou 570228, China; lyx15716360352@163.com (Y.L.); ziy2013@163.com (Y.H.); wangyushan@hainanu.edu.cn (Y.W.); 2 Key Laboratory of Vegetable Biology of Hainan Province, Hainan Vegetable Breeding Engineering Technology Research Center, The Institute of Vegetables, Hainan Academy of Agricultural Sciences, Haikou 571199, China; 3 Xiangyang Academy of Agricultural Sciences, Xiangyang 441057, China; 18327852733@163.com; 4 Lianyungang Academy of Agricultural Sciences, Lianyungang 222000, China

**Keywords:** lignin, reactive oxygen species scavenging, salt tolerance, *Sesuvium portulacastrum*, *SpC3H* gene

## Abstract

Lignin constitutes a fundamental component of plant defense mechanisms against environmental stressors. 4-coumarate 3-hydroxylase (C3H) serves as a pivotal enzyme in lignin biosynthesis. However, its role in the halophyte *Sesuvium portulacastrum* remains uncharacterized. In this study, the *SpC3H* gene was cloned, and subsequent sequence alignment and phylogenetic analyses revealed the highest similarity (57.14%) with BvC3H from *Beta vulgaris*, exhibiting the closest evolutionary relationship with *Beta vulgaris* and *Spinacia oleracea* C3H protein. Quantitative real-time polymerase chain reaction demonstrated that *SpC3H* expression was markedly upregulated in both roots and leaves of *S. portulacastrum* under 800 mM NaCl treatment. Root expression peaked at 48 h (25.3-fold), whereas leaves displayed dual expression maxima at 12 h (7.9-fold) and 72 h (10.7-fold). Subcellular localization assays confirmed cytoplasmic distribution. Heterologous expression in *Arabidopsis thaliana* indicated that transgenic lines exhibited enhanced growth performance, higher fresh weight, and elevated lignin contents relative to wild-type plants under salt stress, accompanied by reduced reactive oxygen species (ROS) accumulation and lower relative electrical conductivity. Furthermore, activities of superoxide dismutase and peroxidase, together with expression of lignin biosynthesis-associated and antioxidant enzyme genes, were markedly elevated. Collectively, these findings establish that SpC3H confers salt tolerance by promoting lignin biosynthesis and activating antioxidant defenses to eliminate ROS, thereby providing a theoretical foundation for genetic improvement of plant salt tolerance.

## 1. Introduction

Soil salinization has progressively intensified as a consequence of global climate change and inappropriate anthropogenic activities (e.g., excessive irrigation, improper management, and fertilizer misuse), emerging as a significant environmental challenge to food security and ecological stability [1,2]. Salt stress hinders water transport in plants, compromises membrane integrity, disrupts ion homeostasis, and triggers excessive accumulation of reactive oxygen species (ROS), ultimately resulting in oxidative injury and metabolic imbalance [3]. To withstand salt stress, plants have developed intricate regulatory strategies, including activation of osmotic adjustment pathways (e.g., proline and betaine biosynthesis) to sustain cellular turgor pressure, along with the induction of enzymatic antioxidant defenses [superoxide dismutase (SOD), catalase (CAT), ascorbate peroxidase (APX), etc.] and non-enzymatic scavenging systems (ascorbic acid, glutathione, etc.) to eliminate ROS, thereby mitigating salt-induced damage to crop development [4,5].

Lignin represents a principal constituent of the secondary cell wall in plant xylem, which imparts the mechanical strength necessary for cellular support and effective transport [6]. Therefore, lignin is indispensable for facilitating the translocation of water and minerals through the xylem and serves a vital function in overall plant growth and development [7]. Additionally, lignin has been shown to participate actively in responses to high salinity stress [8]. Numerous studies have indicated that the upregulation of lignin biosynthesis-related genes enhances lignin deposition and cell wall fortification, thereby conferring resistance to stress-induced damage [9,10]. Within the lignin biosynthetic pathway, the transcriptional regulation of structural genes—including 4-hydroxycinnamate: CoA ligase (*4CL*), cinnamoyl CoA reductase (*CCR*), cinnamyl alcohol dehydrogenase (*CAD*), 4-hydroxycinnamoyl CoA: shikimate/quinate hydroxycinnamoyltransferase (*HCT*), *p*-coumarate 3-hydroxylase (*C3H*), caffeate/5-hydroxyferulate 3-O-methyltransferase (*COMT*), caffeoyl CoA 3-O-methyltransferase (*CCoAOMT*), and ferulate 5-hydroxylase/coniferaldehyde 5-hydroxylase (*F5H*)—has been found to correlate strongly with lignin accumulation in plant tissues [11,12,13].

Cytochrome P450s (CYP450s) constitute a superfamily of genes encoding monooxygenases that catalyze diverse and complex reactions within secondary metabolism in plants, thereby contributing to numerous biological processes, including growth regulation and responses to abiotic stresses [2]. Within the lignin biosynthetic pathway, *p*-coumarate 3-hydroxylase (C3H, belonging to the CYP98A subfamily), as a member of the CYP450 monooxygenase family, catalyzes hydroxylation at the C3 position, converting *p*-coumaroyl shikimate/quinate to caffeoyl shikimate/quinate [14]. This enzyme participates in the formation of coniferyl alcohol and sinapyl alcohol [15,16], thereby directly influencing the ratio of H-type to G/S-type lignin monomers as well as the overall polymerization pattern. Recent investigations have demonstrated that CYP450 family genes can enhance salt tolerance through multiple regulatory pathways. For instance, AtCYP94B3 and AtCYP86B1, regulated by the WRKY9 transcription factor, control suberin biosynthesis and thereby modulate salt tolerance in *Arabidopsis thaliana* seedlings [17]. In *Oryza sativa*, OsCYP71D8L maintains growth equilibrium under salt stress by coordinating gibberellin and cytokinin homeostasis [18]. Similarly, TaCYP81D5 in *Triticum aestivum* confers salt tolerance during both seedling and reproductive stages by enhancing ROS scavenging efficiency [19]. Moreover, *A. thaliana* lines heterologously expressing *Panax ginseng PgCYP736B* exhibited reduced H_2_O_2_ accumulation, increased carotenoid content, and activation of abscisic acid biosynthetic genes, suggesting that CYP450s may regulate salt tolerance by integrating antioxidant defenses with hormone signaling networks [20].

*Sesuvium portulacastrum* L., a perennial succulent herb of the genus *Sesuvium* in the family Aizoaceae, exhibits remarkable tolerance to salinity, drought, heavy metals, and other abiotic stresses [21]. Several salt tolerance-related functional genes have been identified in this species, including SpCIPK2, which enhances salt tolerance in transgenic *A. thaliana* by regulating ion homeostasis and antioxidant enzyme activity [22], and SpAQP1, which mitigates salt-induced injury by activating the antioxidant system [23]. Nevertheless, the role of C3H in mediating salt tolerance in *S. portulacastrum* has not been documented. Transcriptome data from previous studies revealed that salt stress markedly induced sustained upregulation of *SpC3H* within the lignin biosynthetic pathway of *S. portulacastrum* [10], yet its precise function under salinity conditions remains unresolved. In this study, the *SpC3H* gene was cloned from *S. portulacastrum*, its expression profile under salt stress was examined by quantitative real-time polymerase chain reaction, and heterologous expression in *A. thaliana* was conducted to verify the salt tolerance of transgenic lines. These findings provide a theoretical framework for subsequent functional investigations of *SpC3H* and support the potential application of such genes in the genetic improvement of plant salt tolerance.

## 2. Results

### 2.1. Multiple Sequence Alignment and Phylogenetic Tree Analysis of SpC3H

**Figure 1:**
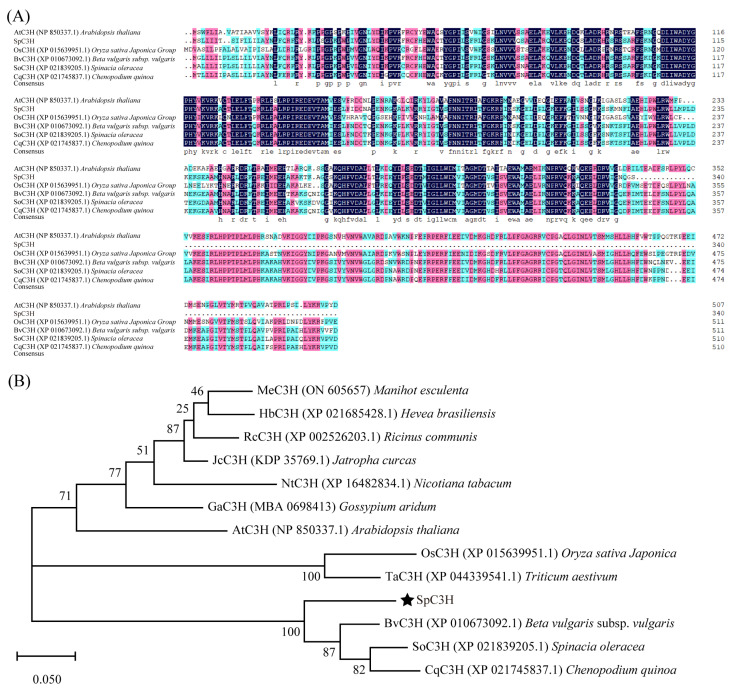
Sequence analysis of SpC3H protein: (**A**) Sequence alignment of C3H proteins from different species. (**B**) Phylogenetic tree analysis of C3H protein. The star represents the SpC3H protein.

In a previous transcriptome analysis of *Sesuvium portulacastrum* under salt stress, *SpC3H* (transcript_HQ_Sp_Three_transcript60205/f2p0/2176) was found to be markedly upregulated in response to salt induction [10]. The *SpC3H* gene was cloned from *S. portulacastrum* cDNA, and multiple sequence alignment of C3H proteins from *S. portulacastrum*, *Oryza sativa*, *Spinacia oleracea*, *Beta vulgaris*, *Chenopodium quinoa*, and *Arabidopsis thaliana* were analyzed using DNAMAN 8.0. The analysis indicated that the SpC3H protein exhibited the highest similarity to BvC3H from *Beta vulgaris*, with 57.14% identity. This was followed by *S. oleracea* and *C. quinoa*, with similarities of 56.37% and 56.18%, respectively, whereas SpC3H shared 42.28% and 37.84% similarity with AtC3H and OsC3H, respectively (Figure 1A).

To further elucidate the evolutionary relationships of SpC3H with other plant species, phylogenetic analysis was performed in MEGA X using amino acid sequences encoded by *C3H* genes from *S. portulacastrum*, *O. sativa*, *A. thaliana*, *Nicotiana tabacum*, *B. vulgaris*, *S. oleracea*, *C. quinoa*, *Manihot esculenta*, *Ricinus communis*, *Hevea brasiliensis*, *Jatropha curcas*, *Triticum aestivum*, and *Gossypium aridum* (Figure 1B). The analysis indicated that BvC3H, SoC3H, and CqC3H shared the closest phylogenetic relationships with SpC3H, whereas more distant relationships were observed with woody species such as MeC3H, HbC3H, RcC3H, and JcC3H.

### 2.2. Expression Analysis of the SpC3H Gene in Response to Salt Stress

**Figure 2:**
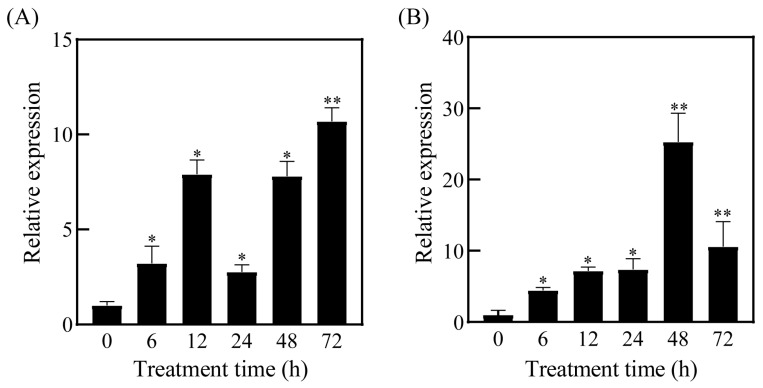
Expression patterns of *SpC3H* under salt stress: (**A**) Expression levels of *SpC3H* in leaves of *S. portulacastrum* following treatment with 800 mM NaCl. (**B**) Expression levels of *SpC3H* in roots of *S. portulacastrum* under 800 mM NaCl treatment. Time points are indicated as follows: 0 h represents the untreated control, and 6, 12, 24, 48, and 72 h represent samples collected after 6, 12, 24, 48, and 72 h of treatment, respectively. Asterisks denote statistically significant differences (* *p* < 0.05; ** *p* < 0.01).

To evaluate the transcriptional response of *SpC3H* under salt stress, RT-qPCR was conducted using seedlings exposed to 800 mM NaCl. The analysis showed that *SpC3H* was upregulated in both roots and leaves of *S. portulacastrum* (Figure 2). In leaves, two expression peaks were observed within 72 h, occurring at 12 h and 72 h, with transcript levels reaching 7.9- and 10.7-fold relative to the initial level, respectively (Figure 2A). In roots, *SpC3H* expression increased rapidly following salt treatment and peaked at 48 h, approximately 25.3-fold higher than the initial level (Figure 2B). These results indicate that *SpC3H* expression is strongly induced by salt stress.

### 2.3. Subcellular Localization of SpC3H

**Figure 3:**
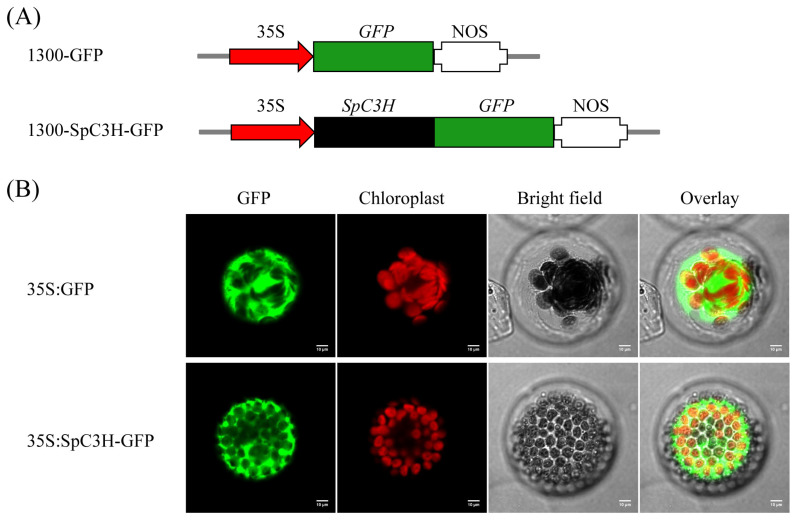
Localization of the SpC3H protein in plant cells: (**A**) Vectors for SpC3H subcellular localization analysis. (**B**) SpC3H localization in cells. *Arabidopsis* mesophyll protoplasts were transformed with the 1300-GFP and 1300-SpC3H-GFP plasmids, and GFP fluorescence was assessed via confocal microscopy. The results are presented from left to right: GFP fluorescence (first column), chloroplast autofluorescence (second column), and bright-field images (third column), with the overlays of these images displayed in the fourth column. Scale bar = 10 μm.

The SpC3H coding sequence was inserted into the pCAMBIA1300-GFP vector to generate a fusion construct with GFP. The recombinant plasmid pCAMBIA1300-*SpC3H*-*GFP* and the empty pCAMBIA1300-*GFP* vector were introduced into *A. thaliana* protoplasts via *Agrobacterium*-mediated transformation. Confocal microscopy revealed fluorescence signals indicating that SpC3H was localized in the cytoplasm (Figure 3).

### 2.4. Heterologous Expression of SpC3H Enhances Salt Tolerance in Transgenic A. thaliana

**Figure 4:**
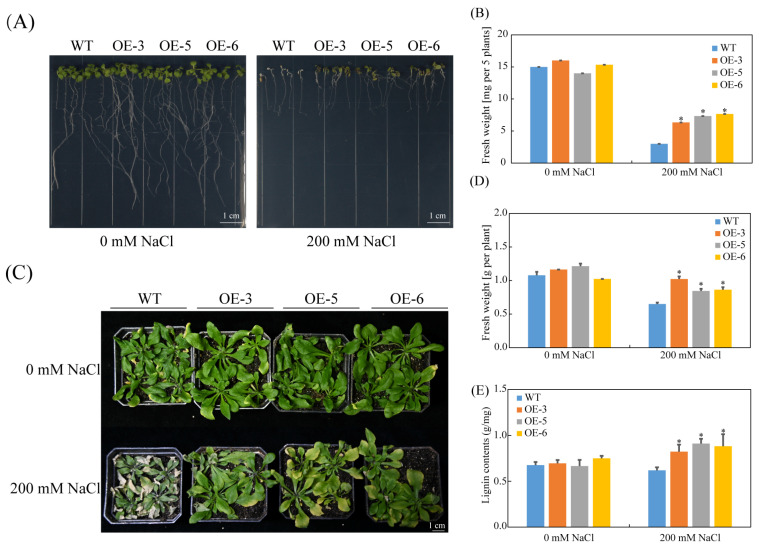
Phenotypes of *SpC3H* transgenic *Arabidopsis* under salt stress: (**A**) Phenotypic analysis of *Arabidopsis* plants grown on 1/2 MS medium under salt stress conditions. (**B**) Fresh weight of the plants shown in (**A**). Data represent the mean ± SD of 12 replicates. (**C**) Phenotypes of *Arabidopsis* grown in soil under salt stress conditions. (**D**) Fresh weight of the plants shown in (**C**). Data represent the mean ± SD of six replicates. (**E**) Lignin contents in different transgenic lines following salt treatment. Data are expressed as mean ± SE from five biological replicates. Asterisks (*) denote statistically significant differences compared to wild-type (WT) controls at *p* < 0.05. WT: wild-type plants; OE-3: *SpC3H*-transgenic line 3; OE-5: *SpC3H*-transgenic line 5; OE-6: *SpC3H*-transgenic line 6. Scale bar = 1 cm.

To investigate the function of *SpC3H*, the recombinant plasmid pCAMBIA1300-*SpC3H* was introduced into *A. thaliana* via *Agrobacterium*-mediated transformation. After selecting HygB-resistant plants on MS plates, we characterized several of these plants using PCR (Figure S1A) and confirmed their identity through semi-quantitative RT-PCR verification (Figure S1B). Three independent transgenic lines exhibiting relatively high expression levels of *SpC3H*, specifically OE-3, OE-5, and OE-6, were selected for subsequent experiments. After 9 days of cultivation on 1/2 MS medium comprising 0 or 200 mM NaCl, seedlings on salt-free medium exhibited vigorous growth with dark green leaves. At 200 mM NaCl, growth inhibition was observed in all plants. However, transgenic lines displayed superior performance relative to WT, with fresh weight markedly higher than that of WT (Figure 4A,B).

To further assess the response of *SpC3H* to salt stress, 8-day-old uniformly grown seedlings from 1/2 MS medium were transplanted into soil and cultivated for one month before salt treatment. After 10 days of NaCl irrigation, WT leaves exhibited yellowing and wilting, whereas transgenic plants maintained normal growth and showed markedly greater fresh weight than WT (Figure 4C,D). Moreover, lignin contents in leaves of transgenic lines under salt stress were markedly higher than those of WT (Figure 4E), suggesting that SpC3H enhances salt tolerance in *A. thaliana* by promoting lignin biosynthesis.

### 2.5. SpC3H Enhances Salt Tolerance in A. thaliana by Regulating Antioxidant Enzyme Activity to Scavenge ROS Accumulation

**Figure 5:**
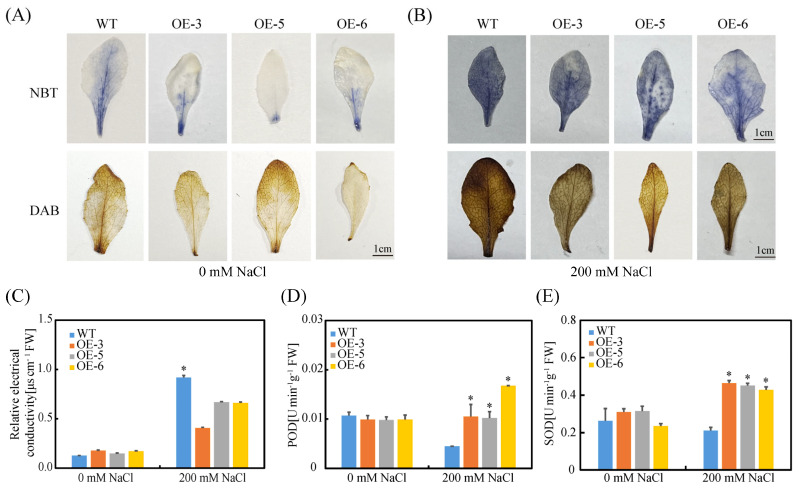
Physiological index determination under salt stress: (**A**) NBT staining of leaves from different *Arabidopsis* genotypes. (**B**) DAB staining of leaves from different *Arabidopsis* genotypes. (**C**) Determination of relative conductivity. (**D**) Measurement of POD activity. (**E**) Measurement of SOD activity. Data are expressed as mean ± SD (n = 3). Asterisks denote statistically significant differences (* *p* < 0.05). WT: wild-type plants; OE-3: *SpC3H*-transgenic line 3; OE-5: *SpC3H*-transgenic line 5; OE-6: *SpC3H*-transgenic line 6. Scale bar = 1 cm.

To clarify the mechanism by which SpC3H modulates ROS metabolism under salt stress, histochemical staining combined with physiological and biochemical analyses was performed. NBT and DAB staining revealed that under normal conditions, both transgenic and WT leaves displayed only slight staining, reflecting low levels of H_2_O_2_ and O_2_^−^. Under salt stress, deeper staining was observed in the leaves of both groups. However, transgenic lines exhibited lighter coloration than WT, indicating that SpC3H overexpression effectively suppressed excessive ROS accumulation (Figure 5A,B). Further measurements showed that the relative conductivity of transgenic lines was markedly lower than that of WT under salt treatment (Figure 5C), suggesting improved maintenance of cell membrane integrity. Moreover, enzymatic assays demonstrated that POD and SOD activities in overexpression lines were markedly elevated compared with WT under salt stress (Figure 5D,E), supporting the conclusion that SpC3H enhances salt tolerance by activating the antioxidant enzyme system to eliminate ROS.

### 2.6. SpC3H Enhanced the Expression of Lignin Biosynthesis Pathway Genes and Antioxidant Enzyme-Related Genes in Transgenic A. thaliana Under Salt Stress

**Figure 6:**
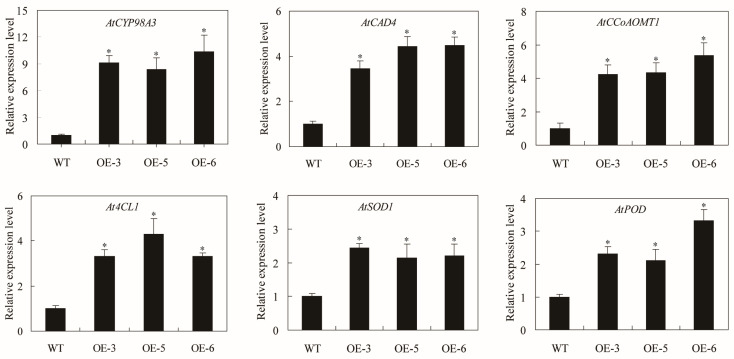
Relative expression analysis of stress-responsive genes was conducted in *SpC3H* overexpressing lines versus the wild type under salt stress. Data are presented as means with standard errors (n = 3). Asterisks (*) denote significant differences at *p* < 0.05 or *p* < 0.01, respectively, compared to WT. WT: wild-type plants; OE-3: *SpC3H*-transgenic line 3; OE-5: *SpC3H*-transgenic line 5; OE-6: *SpC3H*-transgenic line 6.

Given the role of SpC3H in lignin accumulation and ROS scavenging, the expression of stress-related genes under salt stress was examined. The analysis showed that lignin biosynthesis pathway genes, including *AtCYP98A3*, *AtCAD4*, *AtCCoAOMT1*, and *At4CL1*, were upregulated in *SpC3H*-overexpressing lines. Among these, *AtCYP98A3* was markedly upregulated in all three transgenic lines, with transcript levels approximately 9-, 8-, and 10-fold higher than those of WT under salt stress (Figure 6). Antioxidant enzyme-related genes *AtSOD1* and *AtPOD* were also expressed at markedly higher levels in transgenic lines compared with WT.

## 3. Discussion

Lignin, a major component of plant cell walls, enhances cellular mechanical strength. Under salt stress, lignification of root cell walls is intensified, which not only restricts intracellular ion uptake but also increases the rigidity and stability of conductive tissues, thereby improving plant salt tolerance [24]. Salt stress also alters the transcriptional profiles of genes associated with lignin biosynthesis, thereby modulating lignin production as an adaptive response [25]. C3H functions as a precursor enzyme for lignin biosynthesis and represents a key metabolic node linking multiple pathways, exerting a central regulatory role in lignin monomer formation [26,27,28,29]. Specifically, C3H catalyzes hydroxylation at the C-3 position of phenylpropanoid compounds, a reaction essential for determining lignin monomer composition and associated metabolic processes [30]. Suppression of C3H expression has been shown to reduce lignin content by approximately 25% in jute stem segments [30].

Previous research revealed that salt stress induced the expression of lignin biosynthesis pathway genes in *S. portulacastrum* and elevated the levels of sinapic acid, sinapaldehyde, and sinapyl alcohol, with *C3H* markedly upregulated under salt treatment [10]. Consistent with these findings, this study showed that *SpC3H* expression increased in both roots and leaves of *S. portulacastrum* under salt stress, reaching its maximum at 48 h in roots and peaking at 12 h and 72 h in leaves (Figure 2). This temporal expression profile was closely associated with the physiological responses of plants to salinity. As the primary organ sensing salt stress, roots exhibited rapid *SpC3H* induction, likely representing an early response that triggers salt tolerance mechanisms and accelerates protein synthesis to mitigate stress. The biphasic expression observed in leaves may indicate complex regulatory strategies at different developmental stages. The peak at 12 h could be associated with early defense responses, during which salt-tolerant plants adapt to salt stress, followed by a return to lower expression levels. In contrast, the peak at 72 h may correspond to the establishment of long-term adaptation. Previous studies have shown that plants coordinately activate multiple genes in different tissues and at distinct time points under salt stress [3]. This expression pattern suggests the presence of a complex transcriptional regulatory mechanism upstream of SpC3H. However, the specific upstream molecular mechanisms driving its spatiotemporal expression remain unclear. We speculate that salt stress signals may activate the *SpC3H* promoter through transcription factors such as MYB and NAC. For instance, within the lignin synthesis regulatory network, NAC-MYB has been recognized as a core cascade [31]. In *Arabidopsis*, certain NAC transcription factors are considered pivotal for regulating lignin synthesis [32]. OsNAC5 enhances drought resistance in rice by increasing lignin content in the roots [33]. Additionally, MYB transcription factors play a significant role in the transcriptional regulation of lignin. In *Arabidopsis*, MYB58 and MYB63 positively regulate cell wall formation by promoting lignin synthesis [34]. As a transcriptional activator of lignin synthesis in poplar, PtoMYB92 promotes cell wall formation [35]. CmMYB15 regulates lignin biosynthesis in chrysanthemum [36]. Studies have shown that CsMYB330 and CsMYB308 enhance fruit lignification by regulating the expression of the *Cs4CL1* gene in sweet oranges [37]. To further investigate the mechanism by which SpC3H regulates plant salt tolerance, future research should analyze the *SpC3H* promoter sequence to determine whether it contains *cis*-acting elements related to salt stress responses, such as ABA response elements (ABRE) [38], thereby elucidating the molecular pathway of its transcriptional activation.

C3H regulates lignin biosynthesis by modulating the transcription of key genes in the lignin synthesis pathway. In *A. thaliana* overexpressing *PbC3H1*, transcript levels of major lignin biosynthesis genes, including *AtPAL*, *AtC4H*, *At4CL*, *AtHCT*, *AtC3H*, *AtCCOMT*, *AtCCR*, *AtCOMT*, *AtCAD4*, and *AtCAD5*, were markedly elevated relative to WT [39]. Under salt stress, SpC3H enhanced the tolerance of transgenic *A. thaliana* and increased lignin content in leaves (Figure 4). RT-qPCR analysis further demonstrated that *SpC3H* upregulated the transcription of secondary wall formation genes *AtCYB98A3*, *AtCAD4*, *AtCCoAOMT1*, and *At4CL1* in transgenic *Arabidopsis thaliana* (Figure 6), suggesting that SpC3H may contribute to salt tolerance by strengthening secondary wall formation in *A. thaliana*. These results suggest that SpC3H may play a crucial regulatory role under salt stress. Its expression can positively regulate multiple downstream genes, thereby synergistically enhancing secondary cell wall formation.

**Figure 7:**
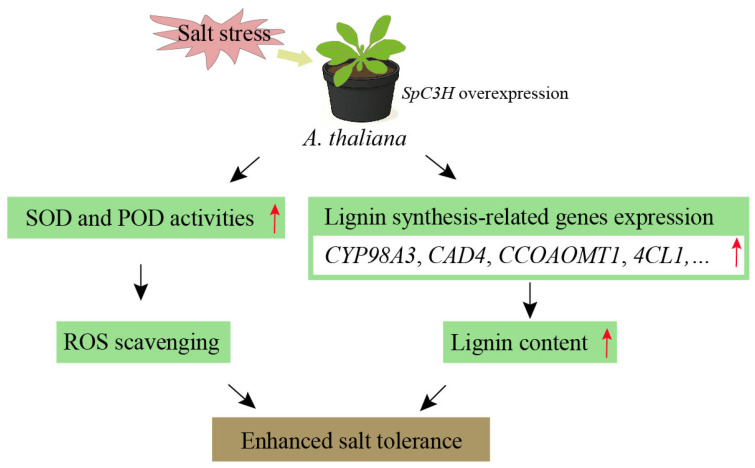
Working model of SpC3H in regulating salt tolerance through ROS scavenging and lignin accumulation. Overexpression of *SpC3H* in transgenic *Arabidopsis* resulted in the upregulation of genes associated with antioxidase system and lignin biosynthesis, thereby scavenging ROS and accumulating lignin in leaves.

Salt stress rapidly induces excessive ROS accumulation, including H_2_O_2_, superoxide, and hydroxyl radicals (OH), which disrupt redox homeostasis and inflict oxidative damage on plant cells [40]. At elevated concentrations, ROS trigger cellular injury through lipid peroxidation, protein oxidation, and nucleic acid damage [41]. SOD constitutes the primary defense against ROS by catalyzing the dismutation of O_2_^−^ into H_2_O_2_ and O_2_, after which H_2_O_2_ is further decomposed into H_2_O and O_2_ by POD and CAT [42,43,44]. In this study, SOD and POD activities in transgenic lines were markedly higher than those in WT under salt stress, while H_2_O_2_ and O_2_^−^ accumulation were effectively suppressed, and relative conductivity was reduced (Figure 5C–E), suggesting improved maintenance of cell membrane integrity. These results indicate that the salt tolerance mechanism mediated by SpC3H involves not only lignin biosynthesis but also activation of the antioxidant defense system (Figure 7). Under salinity, excessive ROS accumulation induces oxidative injury, whereas SpC3H coordinates lignin production with antioxidant defense, thereby enabling efficient ROS scavenging, mitigating oxidative damage, and preserving cell wall structural stability, ultimately enhancing salt tolerance. This mechanism aligns with previous findings that CYP450 family genes confer salt tolerance through diverse pathways. For instance, AoCYP94B3 and AoCYP86B1 from *Avicennia officinalis* promote suberin deposition to restrict Na^+^ transport to aerial tissues and simultaneously participate in ROS metabolism regulation [17]. Previous studies have demonstrated that NAC transcription factors can concurrently regulate genes associated with lignin synthesis and antioxidant enzymes involved in ROS scavenging [45]. Nevertheless, the molecular interactions between these two systems in the salt-tolerant mechanisms of the halophyte *S. portulacastrum* require further investigation.

Although this study clarified the multifaceted roles of *SpC3H* in salt tolerance, the upstream regulatory mechanisms controlling its expression remain unresolved. How salt stress signals are transduced to activate *SpC3H* expression, and whether specific transcription factors or signaling pathways are implicated, requires further investigation. Moreover, the synergistic regulation between lignin biosynthesis and antioxidant defense systems needs to be elucidated, as molecular evidence is still insufficient to explain how these systems interact to enhance plant salt tolerance. Future studies could employ gene-editing technologies to precisely modify *SpC3H* and its upstream or downstream genes, combined with multi-omics approaches such as transcriptomics, proteomics, and metabolomics, to comprehensively dissect the molecular regulatory networks of SpC3H in salt tolerance, thereby providing a stronger theoretical basis for the genetic improvement of plant salt tolerance.

## 4. Materials and Methods

### 4.1. Plant Materials and Treatment

*Sesuvium portulacastrum* was cultivated at the experimental base of Hainan University and propagated hydroponically. Healthy, morphologically uniform cuttings (retaining the apical four stem nodes with the basal two pairs of leaves removed) were selected, rinsed, and positioned in hydroponic cultivation slots with planting cotton. Plants were grown in 1/2 Hoagland nutrient solution under natural light at 25 ± 2 °C. Oxygen concentration in the solution was maintained by an oxygen pump, and the solution was renewed every 5 days. After 10 days of cultivation, plants exhibiting uniform growth were subjected to salt stress by supplementing NaCl into the nutrient solution to a final concentration of 800 mM. Six treatment time points (0, 6, 12, 24, 48, and 72 h) were established, with three biological replicates for each. Leaf and root tissues were harvested separately, immediately frozen in liquid nitrogen, and stored at −80 °C for RNA extraction.

### 4.2. Sequence Alignment

To investigate the evolutionary characteristics of SpC3H, multiple sequence alignment was conducted with ClustalW, and a phylogenetic tree of C3H proteins from diverse species was generated using the maximum likelihood method (Bootstrap = 1000) in MEGA X.

### 4.3. Gene Cloning and Vector Construction

Total RNA was isolated from root samples subjected to salt stress using the RNAprep Pure Plant kit (Tiangen Biotech, Beijing, China, DP441). After DNase I digestion, complementary DNA (cDNA) was synthesized by reverse transcription with the PrimeScript RT reagent kit containing gDNA Eraser (TaKaRa, Dalian, China, RR047A). The resulting cDNA was pooled and used as the template to amplify the coding DNA sequence of the *SpC3H* gene with primers SpC3H-1300-F and SpC3H-1300-R (Table S1). The amplified fragment was ligated into the pCAMBIA1300 plant expression vector at the *Sal* I and *Kpn* I restriction sites, yielding the recombinant plasmid pCAMBIA1300-*SpC3H* for subsequent genetic transformation assays.

### 4.4. Subcellular Localization

The *SpC3H* gene (excluding the stop codon) was amplified from pooled cDNA samples using primers SpC3H-1300-F and SpC3H-1300-GFP-R (Table S1). After double digestion with *Sal* I and *Kpn* I, the amplified product was ligated into the pCAMBIA1300-GFP vector and fused in-frame with GFP, generating the recombinant plasmid pCAMBIA1300-*SpC3H*-*GFP*. The plasmid was introduced into *Arabidopsis thaliana* protoplasts according to the method described by An et al. [46]. Subcellular localization of GFP fluorescence was examined using confocal microscopy, with the empty pCAMBIA1300-GFP vector used as a control.

### 4.5. Gene Expression Analysis

To assess the expression profile of the *SpC3H* gene under salt stress, fluorescent quantitative primers SpC3H-qRT-F and SpC3H-qRT-R were designed (Table S1). Quantitative real-time polymerase chain reaction (RT-qPCR) was carried out using ChamQ™ Universal SYBR qPCR Master Mix (Vazyme, Nanjing, China, 7E280C8), with all procedures performed per the supplier’s protocols. For each sample, three biological replicates were established, and each biological replicate contained three technical replicates. Relative expression levels were determined by the 2^−△△CT^ method, with *SpGAPDH* employed as the internal reference gene [22].

### 4.6. Transformation of Arabidopsis thaliana

The recombinant plasmid pCAMBIA1300-*SpC3H* was introduced into *Agrobacterium* GV3101 and delivered to Biorun Co., Ltd. (Wuhan, China) for transformation of *A. thaliana* (Col-0). T1 seeds were surface-sterilized and germinated on 1/2 Murashige and Skoog (MS) medium comprising 50 mg/L hygromycin B (HygB), followed by cultivation at 22 °C under long-day conditions (16 h light/8 h dark). Resistant seedlings were selected, and genomic DNA was extracted using the TransDirect Plant Tissue PCR Kit (TransGen Biotech, Beijing, China, AD301-01). Transgenic-positive lines were confirmed through PCR amplification using the SpC3H-1300-F and SpC3H-1300-R primers for the *SpC3H* gene, as well as the Hyg-F and Hyg-R primers (Table S1) for the *HygB* gene. Concurrently, RNA was extracted from the seedlings of putative transgenic plants and non-transformed control plants utilizing the RNAprep Pure Plant kit (Tiangen Biotech (Beijing) Co., Ltd., Shanghai China, DP441). First-strand cDNA was synthesized using the PrimeScript RT reagent kit (TaKaRa, Dalian, China, RR047A) and subsequently employed as a template for semi-quantitative RT-PCR analysis with gene-specific primers SpC3H-semi-RT-F and SpC3H-semi-RT-R (Table S1). Positive plants were self-crossed to generate the T2 generation, and single-copy insertion lines were identified based on a 3:1 resistance segregation ratio. Homozygous T3 lines were subsequently employed for experiments.

### 4.7. Salt Stress Treatment

Plate-based salt tolerance assay: Surface-sterilized wild-type (WT) and transgenic *A. thaliana* seeds (T3 generation) were vernalized at low temperature for 48 h, then germinated on 1/2 MS medium for 4 days, and then transferred to 1/2 MS comprising 0 or 200 mM NaCl and cultured vertically for another 9 days. Photographs were taken, and primary root length was quantified using ImageJ (v1.53).

Soil-based salt tolerance assay: Eight-day-old seedlings grown on 1/2 MS medium were transplanted into substrate (vermiculite/soil = 3:1) and cultivated under normal conditions for 4 weeks. Plants were subsequently irrigated with 200 mM NaCl solution for 10 days, after which fresh weight and survival rate were recorded. Three biological replicates were conducted for each assay.

### 4.8. Determination of Physiological Indicators

Lignin content in leaves was quantified using the reagent kit (MZS-1-G, Suzhou Keming Biotechnology Co., Ltd., Suzhou, China) per the supplier’s protocol. Nitroblue tetrazolium chloride (NBT) and 3,3′-diaminobenzidine (DAB) staining were applied to assess H_2_O_2_ and O_2_^−^ accumulation in leaves under salt stress [47,48]. Following the procedure of Dionisio-Sese and Tobita [49], 0.1 g of fresh leaves was immersed in deionized water for 24 h, and initial conductivity (C1) and post-boiling conductivity (C2) were measured with a conductivity meter (DDS-307A). Relative electrical conductivity (REL) was calculated as REL = C1/C2 × 100%. Peroxidase (POD) activity in leaves was determined using the reagent kit (G0107W, Suzhou Grace Biotechnology Co., Ltd., Suzhou, China).

### 4.9. Expression of Lignin Synthesis and Antioxidant Enzyme-Related Genes in Arabidopsis thaliana

To examine the transcriptional response of transgenic *A. thaliana* to salt stress, RT-qPCR was employed to assess the expression of lignin synthesis-related genes *AtCYP98A3* (AT2G40890), *AtCAD4* (AT3G19450), *AtCCoAOMT1* (AT4G34050), and *At4CL1* (At1g51680), as well as antioxidant enzyme-related genes *AtSOD1* (At1g08830) and *AtPOD* (AT3G17070). The *AtACT2* gene was used as the internal reference [22]. Primer sequences for RT-qPCR are provided in Table S1.

### 4.10. Statistical Analysis

All experiments were performed in three biological replicates unless otherwise stated. Statistical analysis was carried out utilizing Excel 2019, and statistical differences were assessed utilizing one-way analysis of variance (ANOVA). Data are expressed as means with standard errors, and *p* < 0.05 was regarded as statistically significant.

## 5. Conclusions

In this study, the key gene *SpC3H*, encoding C3H in the lignin biosynthesis pathway, was cloned from the halophyte *S. portulacastrum*. Phylogenetic analysis indicated that SpC3H shared the closest evolutionary relationship with herbaceous species such as *Beta vulgaris*, *Spinacia oleracea*, and *Chenopodium quinoa*, while showing the most distant relationship with woody plants, including *Manihot esculenta* and *Hevea brasiliensis*. *SpC3H* was markedly induced by salt stress and exhibited tissue-specific expression patterns. Expression in roots peaked at 48 h, reflecting an early response of root systems as the primary organ for salt perception, whereas leaf expression showed two peaks at 12 h and 72 h, potentially corresponding to distinct regulatory phases of short-term stress response and long-term adaptation. Subcellular localization confirmed that the SpC3H protein was localized in the cytoplasm. Under salt stress, transgenic *A. thaliana* heterologously expressing *SpC3H* displayed enhanced growth in both plate and soil culture conditions, confirming its positive regulatory role in salt tolerance. Further analyses revealed increased lignin content in transgenic leaves and elevated transcription of key lignin biosynthesis genes, including *AtCYB98A3*, *AtCAD4*, *AtCCoAOMT1*, and *At4CL1*, indicating that SpC3H improves salt tolerance by reinforcing secondary wall formation. Moreover, transgenic lines exhibited markedly higher SOD and POD activities than WT, accompanied by reduced H_2_O_2_ and O_2_^−^ accumulation, lower relative conductivity, and improved maintenance of cell membrane integrity, confirming that SpC3H enhances antioxidant defense to mitigate oxidative damage. In summary, SpC3H enhances salt tolerance through synergistic regulation of lignin biosynthesis and ROS scavenging pathways. This study provides new insight into the molecular mechanisms of salt tolerance and establishes a theoretical framework for applying *SpC3H* in the genetic improvement of crop salt tolerance.

## Data Availability

The original contributions presented in this study are included in the Supplementary Materials. Further inquiries can be directed to the corresponding authors.

## Supplementary Materials

The following supporting information can be downloaded at https://www.mdpi.com/article/10.3390/plants14213347/s1, Table S1: Primers used in this study. Figure S1: Detection of *SpC3H*-transgenic lines. (A) Displays the detection of DNA amplification in the leaves of transgenic plants. The upper panel shows the amplification of the *SpC3H* gene, while the lower panel depicts the amplification of the *HygB* gene. M: DL2000 marker, 1-12: different transgenic lines, P: positive control, WT: wild-type plant. (B) Presents the semi-quantitative RT-PCR results for the detection of *SpC3H* in the leaves of *SpC3H*-transgenic lines. 1-8: different transgenic lines.
