# Supplementary figures and images for: Sesuvium portulacastrum SpC3H Enhances Salt Tolerance of Arabidopsis thaliana by Regulating Lignin Synthesis and Scavenging Reactive Oxygen Species
Source: Plants (Basel). 2025 Oct 31;14(21):3347. doi: 10.3390/plants14213347 (PMC12608668; doi:10.3390/plants14213347)

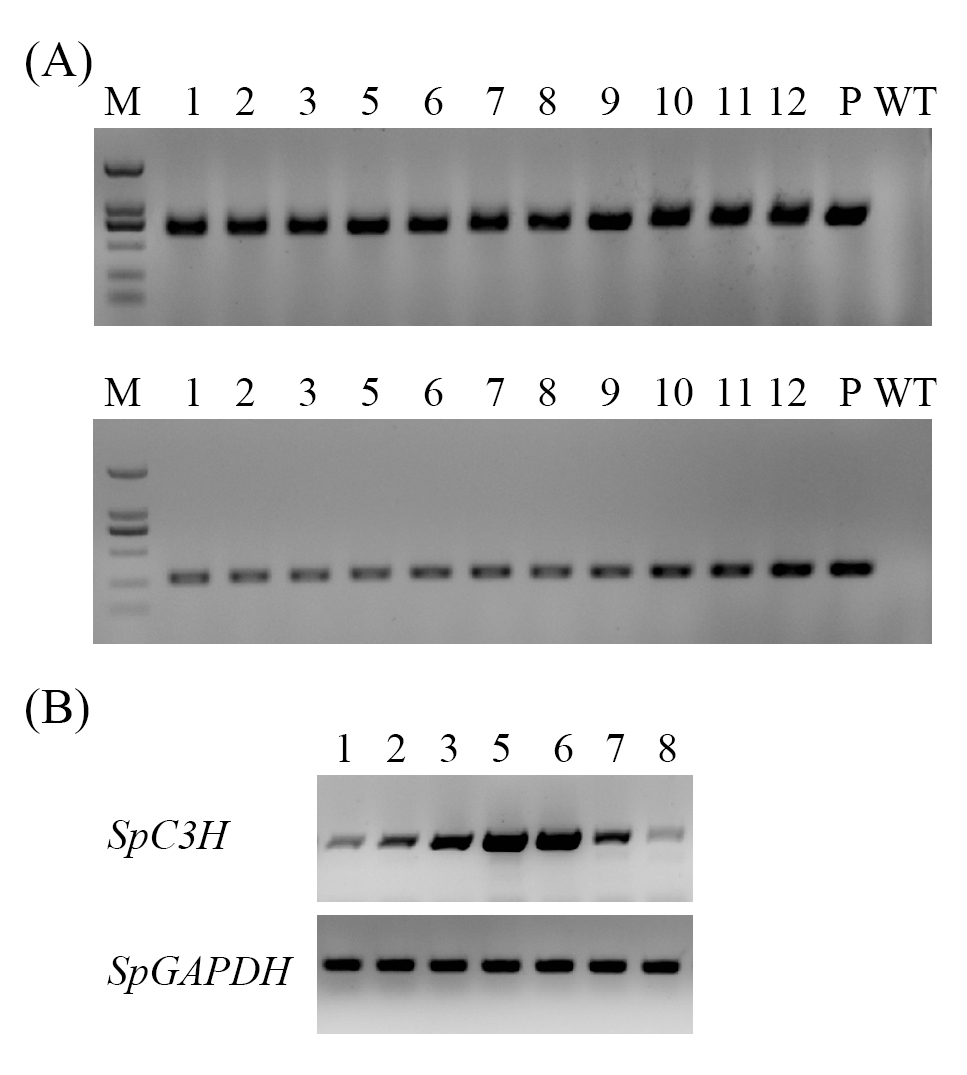

Supplement: Supplementary file 1 [file plants-14-03347-s001.zip › Figure S1.jpg]
